# Serum proBDNF Is Associated With Changes in the Ketone Body β-Hydroxybutyrate and Shows Superior Repeatability Over Mature BDNF: Secondary Outcomes From a Cross-Over Trial in Healthy Older Adults

**DOI:** 10.3389/fnagi.2021.716594

**Published:** 2021-08-20

**Authors:** Jakob Norgren, Makrina Daniilidou, Ingemar Kåreholt, Shireen Sindi, Ulrika Akenine, Karin Nordin, Staffan Rosenborg, Tiia Ngandu, Miia Kivipelto, Anna Sandebring-Matton

**Affiliations:** ^1^Division of Clinical Geriatrics, Center for Alzheimer Research, Department of Neurobiology, Care Sciences and Society, Karolinska Institutet, Stockholm, Sweden; ^2^Division of Neurogeriatrics, Center for Alzheimer Research, Department of Neurobiology, Care Sciences and Society, Karolinska Institutet, Stockholm, Sweden; ^3^Aging Research Center, Department of Neurobiology, Care Sciences and Society, Karolinska Institutet and Stockholm University, Stockholm, Sweden; ^4^Institute of Gerontology, School of Health and Welfare, Aging Research Network - Jönköping (ARN-J), Jönköping University, Jönköping, Sweden; ^5^Ageing Epidemiology (AGE) Research Unit, School of Public Health, Imperial College London, London, United Kingdom; ^6^Theme Inflammation and Aging, Medical Unit Aging, Karolinska University Hospital, Stockholm, Sweden; ^7^Clinical Pharmacology, Karolinska University Hospital, Stockholm, Sweden; ^8^Population Health Unit, Department of Public Health and Welfare, Finnish Institute for Health and Welfare, Helsinki, Finland; ^9^Department of Neurology, Institute of Clinical Medicine and Institute of Public Health and Clinical Nutrition, University of Eastern Finland, Kuopio, Finland; ^10^Research & Development Unit, Stockholms Sjukhem, Stockholm, Sweden

**Keywords:** ketosis, brain-derived neurotrophic factor, proBDNF, signaling metabolites, repeatability, aged humans, β-hydroxybutyrate, cognitive health

## Abstract

**Background:** β-hydroxybutyrate (BHB) can upregulate brain-derived neurotrophic factor (BDNF) in mice, but little is known about the associations between BHB and BDNF in humans. The primary aim here was to investigate whether ketosis (i.e., raised BHB levels), induced by a ketogenic supplement, influences serum levels of mature BDNF (mBDNF) and its precursor proBDNF in healthy older adults. A secondary aim was to determine the intra-individual stability (repeatability) of those biomarkers, measured as intra-class correlation coefficients (ICC).

**Method:** Three of the arms in a 6-arm randomized cross-over trial were used for the current sub-study. Fifteen healthy volunteers, 65–75 y, 53% women, were tested once a week. Test oils, mixed in coffee and cream, were ingested after a 12-h fast. Labeled by their level of ketosis, the arms provided: sunflower oil (lowK); coconut oil (midK); caprylic acid + coconut oil (highK). Repeated blood samples were collected for 4 h after ingestion. Serum BDNF levels were analyzed for changes from baseline to 1, 2 and 4 h to compare the arms. Individual associations between BHB and BDNF were analyzed cross-sectionally and for a delayed response (changes in BHB 0–2 h to changes in BDNF at 0–4 h). ICC estimates were calculated from baseline levels from the three study days.

**Results:** proBDNF increased more in *highK* vs. *lowK* between 0 and 4 h (z-score: β = 0.25, 95% CI 0.07–0.44; *p* = 0.007). Individual change in BHB 0–2 h, predicted change in proBDNF 0–4 h, (β = 0.40, CI 0.12–0.67; *p* = 0.006). Change in mBDNF was lower in *highK* vs. *lowK* at 0–2 h (β = −0.88, CI −1.37 to −0.40; *p* < 0.001) and cumulatively 0–4 h (β = −1.01, CI −1.75 to −0.27; *p* = 0.01), but this could not be predicted by BHB levels. ICC was 0.96 (95% CI 0.92–0.99) for proBDNF, and 0.72 (CI 0.47–0.89) for mBDNF.

**Conclusions:** The findings support a link between changes in peripheral BHB and proBDNF in healthy older adults. For mBDNF, changes differed between arms but independent to BHB levels. Replication is warranted due to the small sample. Excellent repeatability encourages future investigations on proBDNF as a predictor of brain health.

**Clinical Trial Registration:**ClinicalTrials.gov, NCT03904433.

## Introduction

Ketogenic diets—which are based on strict carbohydrate restriction—have been used as a treatment for epilepsy since the 1920's (Kossoff and Cervenka, 2020), and are increasingly investigated in other neurological conditions, including Alzheimer's Disease (AD) (Stafstrom and Rho, 2012; Taylor et al., 2019). Induction of the metabolic state ketosis, characterized by increased circulating levels of the ketone body β-hydroxybutyrate (BHB), is a hallmark of ketogenic diets (Volek et al., 2015). Supplements in the form of ketogenic medium-chain triglycerides (kMCT) (Bach and Babayan, 1982; Norgren et al., 2020b) and exogenous ketones (Poff et al., 2020) provide an opportunity to study ketosis in the absence of carbohydrate restriction. Improved cognitive performance in patients with Mild Cognitive Impairment (MCI) or AD has been reported after intake of kMCT in some (Reger et al., 2004; Henderson et al., 2009; Ota et al., 2019; Fortier et al., 2020), but not in other (Henderson et al., 2020), studies. Ketosis has been attributed as a mediating factor of the positive findings by enhancing brain energetics (Fortier et al., 2019; Cunnane et al., 2020). Potentially, epigenetic brain signaling functions of BHB could be an additional mechanism underlying the observed effects, as such properties of BHB have been established during the past decade (Newman and Verdin, 2017). These signaling functions of BHB include upregulation of the expression of brain-derived neurotrophic factor (BDNF), as shown in mice (Marosi et al., 2016; Sleiman et al., 2016; Hu et al., 2018, 2020). BDNF is one of the most well-studied neurotrophins and is essential for brain function (Lima Giacobbo et al., 2019). Previous exploratory studies in human adults have found some associations between BHB and serum BDNF within a ketogenic diet intervention (Mohorko et al., 2019), and between BHB and plasma BDNF in the context of exogenous ketone ingestion (Walsh et al., 2020). Whether intake of kMCT, and subsequent ketosis, is associated with altered BDNF levels has to our knowledge not been studied. Neither have associations between BHB and BDNF been reported in older adults.

The signaling functions of BDNF are not restricted to the mature form (mBDNF) but encompass also its precursor proBDNF (Lee et al., 2001). More specifically, proBDNF binds to the p75^NTR^ receptor, whereas mBDNF binds preferably to the TrkB receptor (Yang et al., 2017). The functions of mBDNF include promotion of survival and proliferation of neurons and enhancement of memory consolidation, by stimulating long-term potentiation (LTP) in hippocampus. In contrast, proBDNF acts by inducing apoptosis and long-term depression (LTD) (Woo et al., 2005). The brain is dependent on a balance between these counteracting mechanisms in order to fine-tune neurological networks and maintain plasticity (Lu et al., 2005; Diniz et al., 2018). When BDNF is used without prefix in the current article, it either refers to BDNF as a group, or to a reference which does not discriminate between the different forms of the protein. Indeed, a search in the database PubMed generates only 2% of the number of hits for “proBDNF” (or pro-BDNF) compared to “BDNF,” indicating that the literature is limited on studies examining both forms. No study on associations between BHB and proBDNF has been reported so far to our knowledge.

Concentrations of BDNF in peripheral blood have been assumed to serve as a proxy for its function in the brain (Balietti et al., 2018; Rahmani et al., 2019). However, the interpretation of peripheral BDNF is complicated by various factors. First, BDNF is expressed both in the brain and the periphery (Serra-Millàs, 2016; Balietti et al., 2018). Second, it has been a matter of debate whether BDNF can pass the blood-brain-barrier bidirectionally as some animal studies support this assumption (Pan et al., 1998; Alcalá-Barraza et al., 2010; Xie et al., 2020), while an *in vitro* study suggests that BDNF may have poor or null blood-brain barrier penetrability (Pardridge, 2019). Third, levels in serum and plasma appear to be uncorrelated (Nilsson et al., 2020) and have been suggested to represent different pools (Gejl et al., 2019). Very limited evidence is available on comparisons between BDNF levels in human post-mortem brain samples vs. serum, but a recent publication (Bharani et al., 2020) included reports of an inverse correlation between serum and hippocampal proBDNF.

BDNF has been investigated in a number of neurological and psychiatric conditions, including AD (Balietti et al., 2018), MCI (Siuda et al., 2017), Parkinson's Disease (Rahmani et al., 2019), epilepsy (Iughetti et al., 2018), traumatic brain injury (Korley et al., 2016), depression (Molendijk et al., 2014), bipolar disorder (Fernandes et al., 2015a), schizophrenia (Fernandes et al., 2015b), and autism spectrum disorder (Qin et al., 2016). Although significant differences in peripheral BDNF levels between neurologically healthy and subjects with brain disorders have been reported, as reviewed by Lima Giacobbo et al. (2019), the relations are sometimes conflicting or weak (Polacchini et al., 2015). Factors that can influence peripheral BDNF levels include age, gender, medication, lifestyle, depressive symptoms, platelet count and stage of disease (Balietti et al., 2018). Other possible explanations for the inconsistent findings are methodological issues, ranging from circadian rhythms and preanalytical conditions to the specific commercial kits used (Polacchini et al., 2015; Balietti et al., 2018). Additionally, the inconsistencies raise questions on whether peripheral BDNF holds satisfying intra-individual stability to serve as a tool for prediction of disease.

The current article is based on a previously reported clinical trial—with the primary goal to investigate the acute ketogenic effects of ingested medium-chain-triglycerides—which indicated that caprylic acid raise BHB levels in blood 15–30 min after ingestion with a peak after 2–3 h (Norgren et al., 2020b). The primary aim of the current sub-study was to investigate if blood levels of BHB, and changes of those levels, were associated with BDNF levels (proBDNF and mBDNF) in healthy older adults after intake of different fatty acids, including kMCT. Those associations were investigated by comparing arms with differing mean levels of BHB and by direct comparisons between BHB and BDNF on an individual level. Finally, we investigated whether baseline levels of mBDNF and proBDNF are consistent within individuals, when measured under similar conditions on three different study days within a month. This measure of intra-individual stability will be referred to as repeatability.

## Materials and Methods

Details of the study method have been published previously (Norgren et al., 2020a,b), and a summary of details relevant for this article follows here.

### Study Sample

Fifteen healthy volunteers, 53% women, were recruited by advertising in a daily newspaper. Inclusion criteria were age 65–75 years, written informed consent during a screening visit, and daily coffee consumption, as coffee was used as a vehicle. Exclusion criteria were weight <50 kg, current smoking, diagnosed diabetes (type 1 or 2), history of heart disease, history of disease related to internal organs or metabolism, experience of “sensitive gut” or known intolerance to coconut oil or sunflower oil, medication expected to affect glucose- or lipid-metabolism, fasting during the study or one month before, high intensity physical activity more than 3 days/week, dementia, severe psychiatric conditions, Hb < 125 g/L, and participation in a lifestyle intervention during the last 6 months. The sample size was estimated to be sufficient to detect differences of clinical significance for our primary outcome (BHB) with a power of 80 % and alpha = 0.05, based on effect sizes reported in previous studies (Vandenberghe et al., 2017). For the current sub-study, no reference was available to estimate what would constitute a meaningful or expected effect size for changes in BDNF, and the analyses should therefore be considered exploratory.

### Study Design

In a cross-over design, participants following their usual diet were exposed to six different intervention arms, weekly in a randomized order. The wash-out period between the test days was assumed to eliminate any carry-over effects. The test oils were served in a covered cup, mixed with 2.5 dl coffee, containing approximately 170 mg caffeine, and 15 g full-fat cream. Participants were informed that fatty acids from coconut oil and sunflower would be used in the study but were blinded from further details. The current article is based on three of those arms, distributed within the first four weeks, which included measures on BDNF. The arms selected were presumed to represent three different ketone levels: lower (sunflower oil, 30 g; lowK), midrange (coconut oil, 30 g; midK) and higher (caprylic acid, 20 g + coconut oil, 30 g; highK). Ketone levels—measured as BHB, area under the curve (AUC)/time—turned out not to differ significantly between *midK* (0.22 mmol/L) and *lowK* (0.18 mmol/L), but for *highK* (0.45 mmol/L) vs. the other two (Norgren et al., 2020b). The highest BHB peak was 1.3 mmol/L. The MCT (C6-C12) contents of the test oils were 0% for sunflower oil, 100 % for caprylic acid (C8 only) and ≈62% (mainly C12) for coconut oil (Norgren et al., 2020b). The main fatty acid constituents of sunflower oil were C18:2 (48–74%) and C18:1 (14–39%), according to the ranges specified by the manufacturer.

### Sampling Procedure and Laboratory Analyses

Recruitment and data collection took place between August and October 2018. The study was conducted at the Clinical Pharmacology Trial Unit (CPTU) at the Karolinska University Hospital, Huddinge, Sweden. Participants were instructed to keep their usual habits regarding diet and activity. Instructions were given to consume nothing but water after 8 p.m. the day before testing, to not consume alcohol the day before testing, and to avoid physical activity exceeding 20 min stroll in the morning of a study day. Participants arrived at 7:30 a.m. on each study day. During the study session they were at rest, and water was allowed *ad libitum*. A venous catheter was applied for repeated collection of blood. Baseline (T0) blood samples were performed 20–40 min after arrival. Within 5 min after the blood draw, participants received the test drink in a covered cup.

BDNF was measured at T0, 60, 120 and 240 (min). Blood samples were allowed to coagulate at room temperature for 30 min and then centrifuged at +4°C for 10 min at 2,000 × g to separate serum. The collected serum was stored in polypropylene tubes at −80°C until assayed. Protein concentrations of mBDNF and proBDNF were determined using commercially available enzyme linked immunosorbent assay (ELISA) kits (mBDNF: DBD00, R&D Systems, USA; proBDNF: DY1375 DuoSet ELISA and DY008 Ancillary kit, R&D Systems, USA). For mBDNF, samples were diluted 1:20 in reagent diluent RDP6 and the procedure was performed according to the manufacturer's instructions. For proBDNF, minor modifications on the manufacturer's protocol were applied; samples were diluted 1:2 in a PBS buffer containing 10% FBS (Fetal Bovine Serum) and 0.02% Tween-20 and incubated overnight at +4°C. Standards were diluted in the same buffer. All measurements were performed in duplicate and the average was subsequently used for the statistical analyses. Three serum samples were assayed in all plates, to assess the inter-assay variability of each method. Absorbance was obtained at 450 nm with a microplate reader (Tecan Life Sciences, Männedorf, Switzerland). Protein concentrations were calculated from the standard curves with GraphPad Prism 8.3.1 software using a 4PL curve fit. The intra- and inter-assay coefficients of variation (CV%) were <5% and <10% for mBDNF and <5% and <17% for proBDNF, respectively.

Our main ketone outcome, BHB assessed in venous whole blood (BHBv), was measured with a point-of-care meter (Statstrip Xpress^®^) at 12 time points. BHBv was validated against a laboratory assay on plasma BHB (BHBp) at four of those timepoints (Norgren et al., 2020a). These analyses indicated that the agreement between BHBv and BHBp was high (Lin's concordance correlation coefficient of absolute agreement = 0.91). For analyses in the current sub-study, we created a BHB variable equaling the gold standard BHBp when available (T0, 30, 60, 120), and equaling BHBv at timepoints beyond T120. BHBp levels were measured using the Randox D-3 Hydroxybutyrate (Ranbut) reagent kit (Randox, Crumlin, UK).

The study was approved by The Regional Ethical Review Board in Stockholm and complies with the Declaration of Helsinki. The study was registered as a clinical trial on ClinicalTrials.gov, NCT03904433.

### Statistical Analyses

Standardized scores are reported, unless a unit is specified. The z-score represent a relative measure of effect sizes within this sample. BHB and proBDNF were log-transformed for improved normality. Eight percent of the BHB(v) values at T150-240 were zero, as a consequence of having just one decimal reported by the meter used at T>120. For those data points, the value 0.025 was imputed to allow log-transformation (based on the assumption that the true value was in the range 0–0.05). AUC was calculated by the trapezoidal method, and abbreviated nAUC when normalized to T0. Correlations were determined by Pearson's *r*. Results and confidence intervals (CI) are reported with a significance level of *p* < 0.05. The software used for all calculations was STATA 15.

As we did not have one specific hypothesis on whether the BDNF response would be linear, transient, rapid or delayed, we used different outcome variables to exploratory compare the arms: Estimated differences in change from baseline (ΔT0-60, ΔT0-120, ΔT0-240) and baseline values were analyzed in a mixed regression model with *arm* x *time-point* as categorical variables, with individual as a random factor, and robust standard errors. While ΔT0-240 was assumed to capture a potentially delayed response, we additionally analyzed nAUC_T0-240 as a cumulative measure which could represent a potentially transient response, even if the timing of the peak differs between individuals. For comparisons of nAUC between arms, we performed ANOVA for repeated measures, using Huynh-Feldt correction. Subsequent pair-wise *t*-tests for unequal variances were performed. HighK vs. LowK was a prioritized comparison and is therefore reported without adjustment for multiple comparisons (as well as the comparisons with midK for comparability). Associations between BHB and BDNF on an individual level were analyzed in a linear regression model, adjusting for individual. These analyses were made cross-sectionally at the four available time-points. Additionally, a lag-analysis was performed for a potentially delayed association between BHB [*nAUC_T0-120*, representing an important timespan of the ketogenic response (Norgren et al., 2020b)] and BDNF *(*Δ*T0-240*). The analysis methods were chosen after graphically validating acceptable normality of the outcome variables. The *p*-values are transparently reported without adjustment for analyzing four different timeframes, since e.g., Bonferroni correction (using a threshold at *p* = 0.0125) might be too conservative considering expected correlation among the outcomes (Moran, 2003).

Repeatability, i.e., the biological stability of a variable within an individual, was assessed by calculation of Intra Class Coefficients (ICC), which has been described as the most frequently used method for this purpose (Wolak et al., 2012). ICC estimates were calculated based on a single-rating, absolute-agreement, 2-way mixed-effects model (Koo and Li, 2016). Analyses were made on baseline data from the three different study days. To assess discriminability, here defined as the capacity of mBDNF and proBDNF to detect significantly different mean values between individuals, *t*-tests were performed based on the three baseline assessments. We calculated the proportions of *p*-values being <0.05 and <0.001 (Bonferroni corrected) from all (i.e., 105) pair-wise comparisons.

## Results

### Baseline Characteristics, Data Collection and Safety

Baseline characteristics of the participants are reported in Table 1, stratified by sex. The age of the participants was in the range 65–73 years. After observing that age and body mass index (BMI) were higher and more skewed in their distribution among men compared to women, we concluded that it was not meaningful to conduct further stratified analyses since their interpretation would be too ambiguous in this small sample. All 15 participants completed the study. Data collection was complete regarding data points relevant to the current article. Tolerance to the test drinks was in general good, and the few inconveniences reported were mainly related to minor/moderate gastrointestinal events or nausea (Norgren et al., 2020b).

**Table 1:** Baseline characteristics of the participants.

**Variable**		**All (*N* = 15)**	**Women** **(*n* = 8)**	**Men (*n* = 7)**
Age (years)		69.2 ± 2.4	68.6 ± 2.2	69.9 ± 2.6
BMI (kg/m^2^)		23.9 ± 4.0	22.9 ± 2.6	24.9 ± 5.2
HbA1c (mmol/L)		37.2 ± 2.0	37.5 ± 2.1	36.9 ± 2.0
Glucose (mmol/L)	lowK	5.3 ± 0.5	5.2 ± 0.2	5.4 ± 0.7
	midK	5.2 ± 0.6	5.1 ± 0.3	5.3 ± 0.9
	highK	5.2 ± 0.3	5.1 ± 0.3	5.3 ± 0.4
Insulin (mIE/L)	lowK	6.1 ± 3.9	6.2 ± 2.3	6.0 ± 5.5
	midK	5.0 ± 2.9	5.5 ± 2.2	4.3 ± 3.5
	highK	5.5 ± 3.9	6.2 ± 3.8	4.6 ± 4.2
BHB (mmol/L)	lowK	0.09 ± 0.09	0.08 ± 0.04	0.11 ± 0.13
	midK	0.14 ± 0.08	0.13 ± 0.06	0.15 ± 0.09
	highK	0.11± 0.07	0.09 ± 0.04	0.14 ± 0.08
proBDNF (ng/ml)	lowK	1.2 ± 1.8	0.60 ± 0.30	1.8 ± 2.5
	midK	1.3 ± 2.1	0.62 ± 0.29	2.0 ± 3.1
	highK	1.5 ± 3.0	0.58 ± 0.38	2.5 ± 4.3
mBDNF (ng/ml)	lowK	24.8 ± 10.1	25.0 ± 10.7	24.5 ± 10.3
	midK	27.6 ± 9.0	28.0 ± 9.9	27.1 ± 8.7
	highK	29.8 ± 10.6	30.4 ± 10.6	29.1 ± 11.4

*Age, body mass index (BMI) and hemoglobin A1c (HbA1c) were assessed only at study start. BHB: β-hydroxybutyrate; BDNF: brain-derived neurotrophic factor; mBDNF: mature BDNF*.

*Mean values and standard deviations*.

### Description of BHB and BDNF Levels in the Different Arms

**Figure 1:**
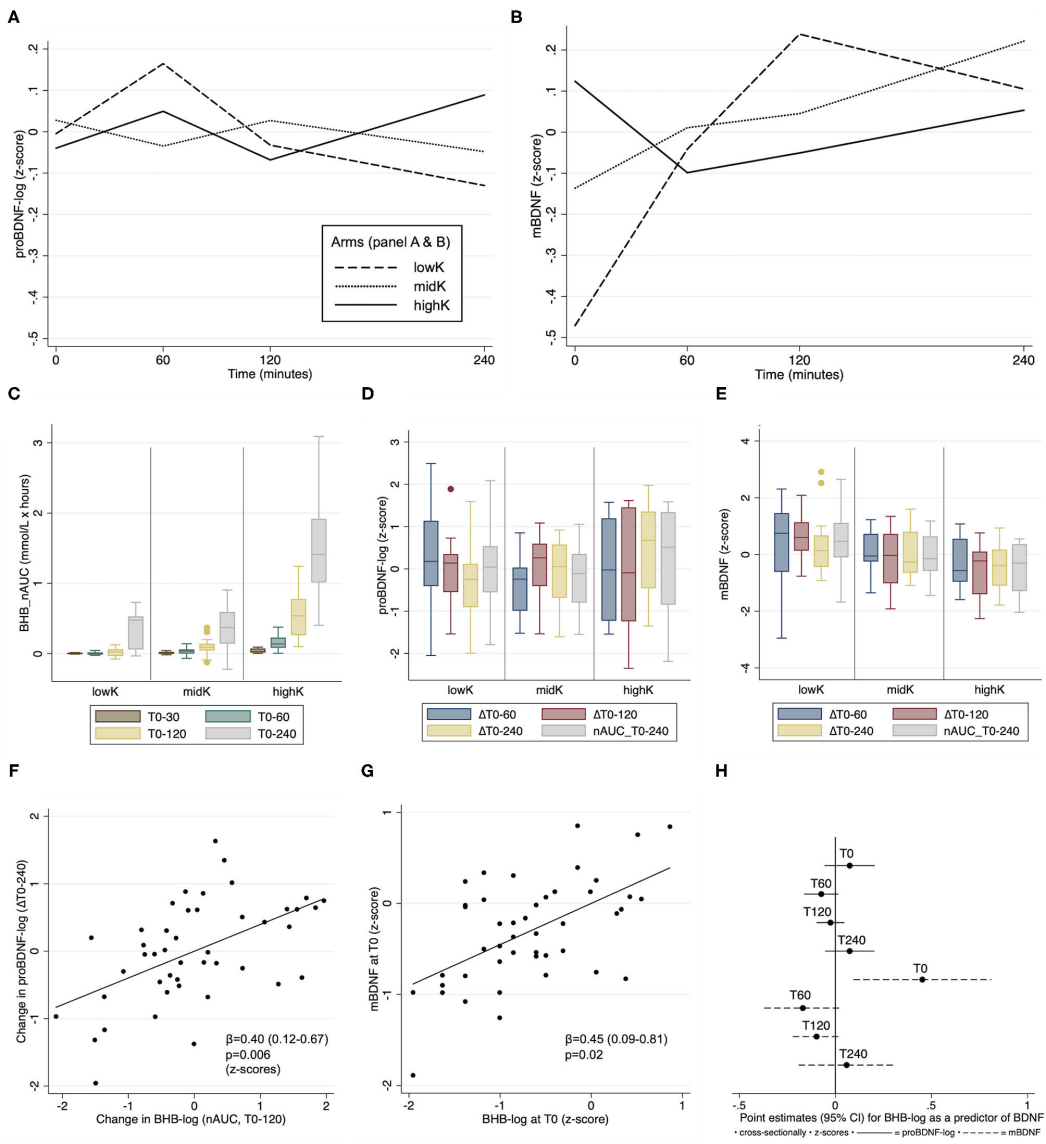
Comparisons of biomarker differences between arms **(A–E)**, and individual associations between BHB and BDNF **(F–H)**. **(A,B)** Profile plots describing the dynamic changes in mean proBDNF-log and mBDNF, respectively. **(C)** The cumulative ketogenic response in arms, measured as nAUC (area under the curve normalized to T0 [min]) for β-hydroxybutyrate (BHB). **(D,E)** Box plots describing the response per arm for proBDNF and mBDNF, respectively. **(F,G)** Partial residual plots showing individual associations between BHB and BDNF outcomes. **(H)** Point estimates of cross-sectional associations between BHB and BDNF on an individual level at different timepoints. BDNF: brain-derived neurotropic factor. Box plots indicate median, interquartile range and maximum/minimum values.

Figures 1A,B include profile plots of mean proBDNF and mBDNF at different time-points per arm (absolute levels reported only descriptively since the main outcome is *change*). Notably, the mean concentration of mBDNF at T0 was significantly lower in *lowK* vs. *highK* (difference 5.0, CI: 1.0 to 9.0 ng/ml, *p* = 0.01, illustrated on a z-scale in Figure 1B). For proBDNF, no such difference between any arms at T0 was observed (*p* > 0.66). The distributions of the cumulative ketogenic response in arms are described in a boxplot (Figure 1C), indicating that BHB levels mainly differed between *highK* and the other two arms, whereas the difference between *lowK* and *midK* was very small.

### Comparisons of the BDNF Changes Between Arms

Table 2 lists the four outcomes for proBDNF and mBDNF, as pair-wise comparisons of means between arms. In summary, for proBDNF statistically significant differences between arms were only observed for the time-variable ΔT0-240, where levels increased more in *highK* compared to the other two arms. In contrast, the comparisons of mBDNF levels indicated a significantly higher increase for *lowK* vs. *highK* for three of the time-variables (Table 2). With a conservative adjustment of the significance level to *p* = 0.0125, considering the four different timeframes, results would still be significant for *proBDNF_*Δ*T0-240, mBDNF_*Δ*T0-120* and *mBDNF_nAUC_T0-240*. Two boxplots describe the distribution of calculated changes in levels of proBDNF (Figure 1D) and mBDNF (Figure 1E) for the four different time-variables.

**Table 2:** Comparisons of the BDNF response between arms.

**Outcome**	**Comparison**	**Coefficient (95% CI)**	***P*-value**
proBDNF-log ΔT0-60 (minutes)	highK vs. lowK	−0.08 (−0.39 • 0.23)	0.62
	highK vs. midK	0.15 (−0.06 • 0.36)	0.15
	midK vs. lowK	−0.23 (−0.47 • 0.01)	0.06
proBDNF-log ΔT0-120	highK vs. lowK	−0.01 (−0.25 • 0.25)	0.99
	highK vs. midK	−0.03 (−0.27 • 0.21)	0.82
	midK vs. lowK	0.03 (−0.19 • 0.25)	0.81
proBDNF-log ΔT0-240	**highK vs. lowK**	**0.25 (0.07 • 0.44)**	**0.007**
	**highK vs. midK**	**0.20 (0.03 • 0.38)**	**0.02°**
	midK vs. lowK	0.05 (−0.11 • 0.21)	0.55
proBDNF-log nAUC_T0-240	highK vs. lowK	0.19 (−0.67 • 1.04)	0.66
	highK vs. midK	0.34 (−0.45 • 1.13)	0.39
	midK vs. lowK	−0.15 (−0.78 • 0.48)	0.62
mBDNF ΔT0-60	highK vs. lowK	−0.65 (−1.45 • 0.15)	0.11
	highK vs. midK	−0.37 (−0.92 • 0.19)	0.19
	midK vs. lowK	−0.28 (−0.97 • 0.40)	0.42
mBDNF ΔT0-120	**highK vs. lowK**	**−0.88 (−1.37 • −0.40)**	**<0.001**
	**highK vs. midK**	**−0.36 (−0.69 • −0.02)**	**0.04°**
	**midK vs. lowK**	**−0.53 • (−1.02 • −0.04)**	**0.03°**
mBDNF ΔT0-240	**highK vs. lowK**	**−0.65 (−1.24 • −0.05)**	0.03°^*^
	highK vs. midK	−0.43 (−0.87 • 0.02)	0.06
	midK vs. lowK	−0.22 (−0.85 • 0.41)	0.50
mBDNF nAUC_T0-240	**highK vs. lowK**	**−1.01 (−1.75 • −0.27)**	**0.01**
	highK vs. midK	−0.51 (−1.13 • 0.10)	0.10
	midK vs. lowK	−0.50 (−1.23 • 0.24)	0.17

*highK = 20 g caprylic acid + 30 g coconut oil; midK = 30 g coconut oil; lowK = 30 g sunflower oil. The comparisons are organized in a way that a positive coefficient can be interpreted as the response variable being higher in the arm with higher mean β-hydroxybutyrate (BHB). Bold text indicate significant results. Standardized coefficients. BDNF: brain-derived neurotrophic factor; mBDNF: mature BDNF; K: ketosis*.

* *Not significant after the exclusion of two outliers. °Not significant with a Bonferroni adjusted p-threshold at 0.0125*.

A sensitivity analysis, excluding an individual (a male of normal weight) whose proBDNF levels (before log-transformation) were consistently in a 10-fold higher range compared to the others, indicated marginally strengthened results in the same direction for *proBDNF_*Δ*T0-240* (highK vs. lowK: *p* = 0.001; highK vs. midK: *p* = 0.01). For *mBDNF_*Δ*T0-240*, an additional analysis excluding two outliers for that variable (visible in Figure 1E) was performed, after which the difference between arms was no longer significant (*p* > 0.16). For comparison, *mBDNF_*Δ*T0-120* (*p* = 0.002) remained significant after exclusion of the same individuals.

### Associations Between Individual Levels of the Ketone BHB and BDNF

A delayed association between BHB (nAUC_T0-120) and proBDNF (ΔT0-240) was observed (β = 0.40, CI: 0.12–0.67, p=0.006, Figure 1F). No such delayed association was observed for mBDNF (β = −0.14, CI: −0.50 to 0.22, *p* = 0.43). At T0, BHB was significantly associated with mBDNF cross-sectionally (β = 0.45, CI: 0.09 to 0.81, *p* = 0.02, Figure 1G), but not with proBDNF (β = 0.07, CI: −0.06 to 0.21, *p* = 0.25). No significant cross-sectional association with BHB for any BDNF marker was observed at any other time-point (Figure 1H).

**Figure 2:**
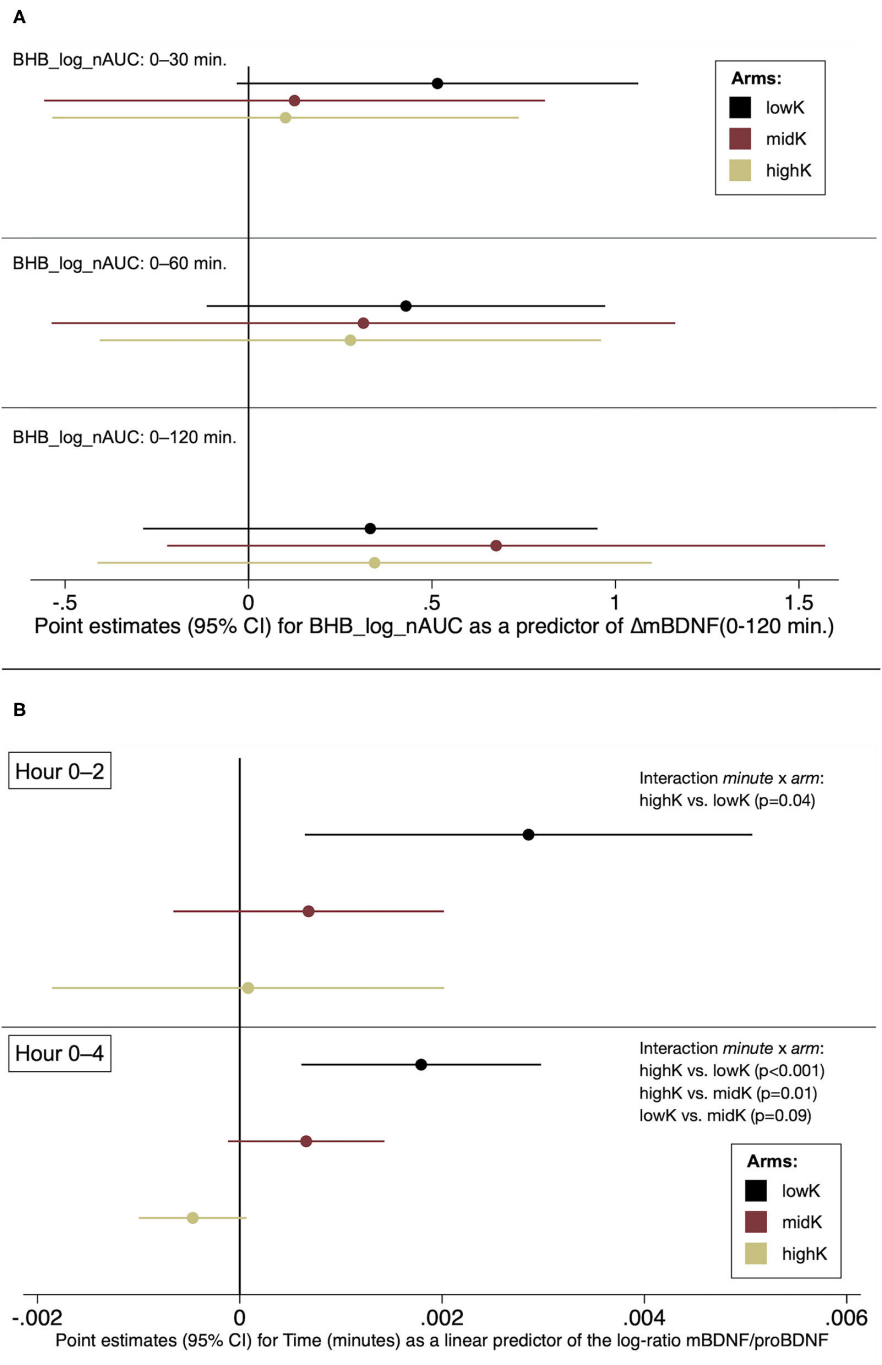
Post-hoc analyses. **(A)** The early ketogenic response as a predictor of change in mBDNF, T0-120 **(B)** Changes in the log-ratio mBDNF/proBDNF by arm.

After observing significant differences between all arms—in a direction opposite to our expectations—for *mBDNF_*Δ*T0-120* (Table 2), a *post-hoc* analysis was performed to investigate if that change in mBDNF was (negatively) associated with the early ketogenic response. However, neither *BHB-log_nAUC* for T0-30, T0-60 nor T0-120 within any arm were associated with *mBDNF_*Δ*T0-120* with a negative point estimate (Figure 2A). Further analyses were performed to explore whether differing conversion rates from proBDNF to mBDNF possibly could underlie the results. Indeed, those analyses indicated that the log-ratio mBDNF/proBDNF increased more in lowK compared to the other arms during the first two h, as well as during the whole 4-h period (Figure 2B).

### Repeatability and Discriminability of proBDNF and mBDNF

**Figure 3:**
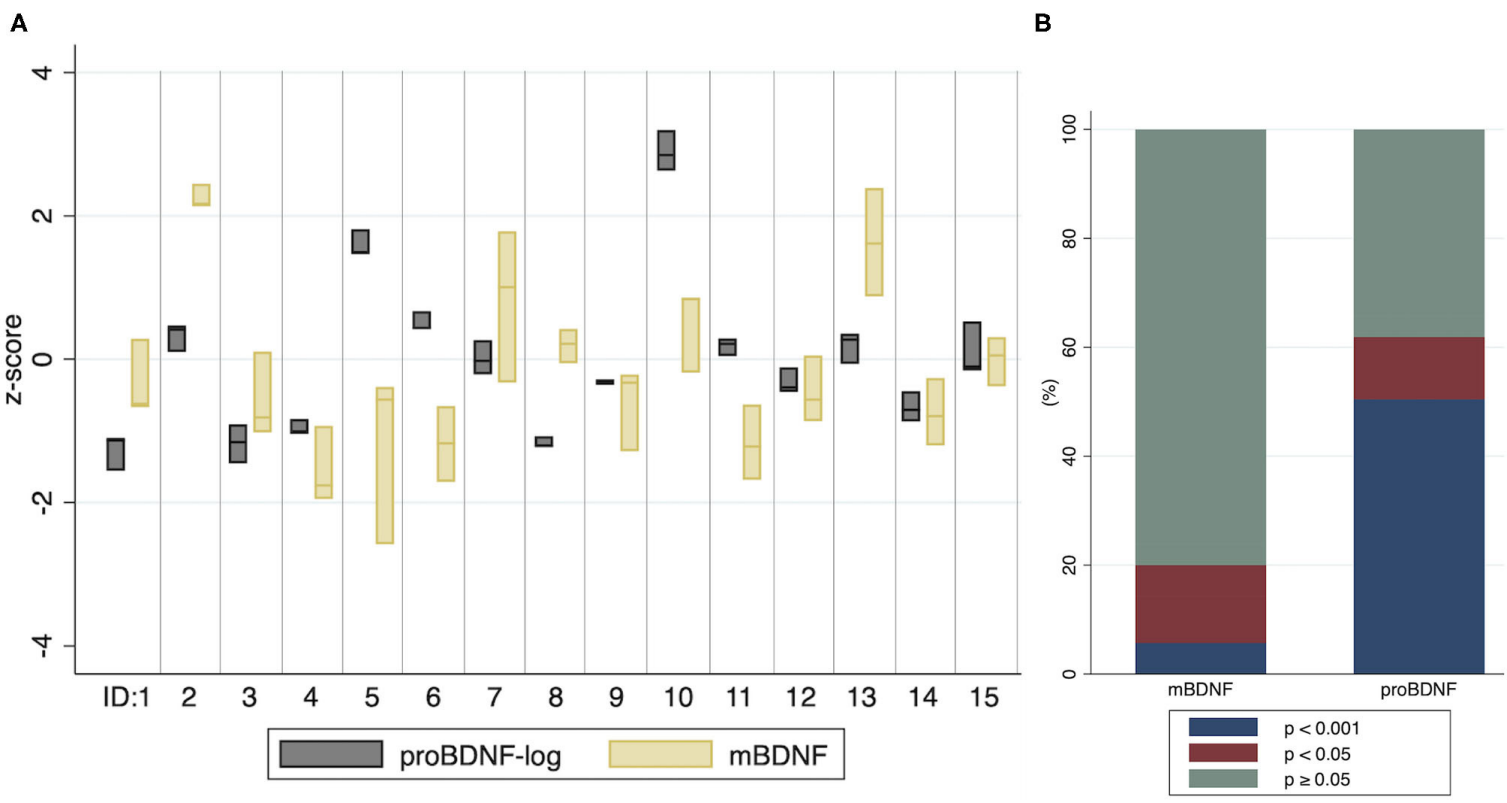
Distribution of proBDNF-log and mBDNF per individual, and the significance of pair-wise comparisons of differences in baseline means **(A)** Values from baseline (T0) at three study days within a month. The boxes indicate minimum, maximum and median values. ID: 1–15 represent individuals. **(B)** The proportion of pair-wise comparisons of individual baseline means, by categories *p* < 0.001, *p* < 0.05 and *p* ≥ 0.05.

The intra- and inter-individual distributions of mBDNF and proBDNF at T0, measured at the three study days, are illustrated by a box-plot (Figure 3A). A visual interpretation suggests higher biological stability in proBDNF compared to mBDNF which is further confirmed by the ICC values reported in Table 3, showing non-overlapping confidence intervals for proBDNF_log (0.92–0.99) and mBDNF (0.47–0.89). For comparison, ICC for all biomarkers are shown with and without log-transformation, even when transformation was not necessary since the original variable was approximately normally distributed. ICC for BHB is reported exploratorily, indicating low repeatability in this range. The labeling of reliability (as a measure of repeatability) in Table 3 was made according to Koo and Li (2016): poor (<0.50), moderate (0.50–0.75), good (0.75–0.90) and excellent (≥0.90).

**Table 3:** Distributions and Intraclass Correlation Coefficients (ICC) for different biomarkers.

**Biomarker**	**Percentiles 10, 25, 50, 75, 90**	**ICC (95% CI)**	**Reliability**
proBDNF	0.24, 0.31, 0.60, 0.97, 2.6 (ng/ml)	0.92 (0.83 • 0.97)	Good–Excellent
proBDNF-log		0.96 (0.92 • 0.99)	Excellent
mBDNF	15, 21, 26, 31, 44 (ng/ml)	0.72 (0.47 • 0.89)	Poor–Good
mBDNF-log		0.57 (0.28 • 0.81)	Poor–Good
totBDNF	15, 22, 27, 33, 48 (ng/ml)	0.74 (0.49 • 0.89)	Poor–Good
totBDNF-log		0.62 (0.33 • 0.83)	Poor–Good
BHBp	0.04, 0.06, 0.09, 0.15, 0.24 (mmol/L)	0.38 (0.08 • 0.69)	Poor–Moderate
BHBp-log		0.30 (0.02 • 0.63)	Poor–Moderate

*Measures from baseline (T0) at three visits within a month. proBDNF: precursor to brain-derived neurotrophic factor (BDNF); mBDNF: mature BDNF; totBDNF: proBDNF + mBDNF; BHBp: plasma β-hydroxybutyrate*.

The correlation between mBDNF and proBDNF-log was moderately positive (*r* = 0.20, *p* < 0.01) and concentrations of mBDNF were ≈50-fold higher compared to proBDNF (Table 3). As a consequence, a very high correlation (*r* = 0.98, *p* < 0.001) was observed between total BDNF (totBDNF = mBDNF + proBDNF) and mBDNF. Exploratory analyses on totBDNF corresponded almost perfectly with mBDNF and are not reported beyond ICC.

A higher discriminability for proBDNF compared to mBDNF was observed by analyzing *p*-values from pair-wise comparisons of differences in individual baseline means from the three study days (Figure 3B). For proBDNF-log, 62% of the pair-wise comparisons were significant (*p* < 0.05), and 50% had a *p*-value < 0.001. For mBDNF, the corresponding proportions were 20% and 6%. For BHB-log, no pair-wise comparison of individual baseline means had a *p*-value < 0.13, suggesting that BHB at these low levels does not substantially discriminate individuals from each other.

## Discussion

In this study we found different BDNF responses between the intervention arms after ingestion of various fatty acids, including kMCT. For proBDNF, the differences between arms corresponded to associations between BHB and proBDNF on an individual level, suggesting ketosis could play a mechanistic role. To our knowledge, this is the first report of associations between BHB and proBDNF in humans, and between BHB and BDNF in healthy older adults. BHB levels at baseline were positively associated with mBDNF but, contrary to our expectations, mBDNF increased more in the low vs. the high ketosis arm for at least two of the outcome variables (while the statistical significance of results in the same direction for another variable relied on the inclusion of two outliers). Since the differing mBDNF responses between arms did not correspond to any individual associations between BHB and mBDNF, those observed results might be explained by factors unrelated to ketosis. A striking finding in our data is proBDNF combining high intra-individual stability with superior inter-individual discriminability compared to mBDNF at the daily baseline, a finding that might facilitate the interpretation of the longitudinal BDNF responses within this study.

In the arm with the highest cumulative levels of the ketone BHB vs. the two other arms we observed a significantly higher increase in proBDNF after four hours—but not after one or two hours—which could be interpreted as a delayed response to the intervention. Upregulation of the *bdnf* gene in response to BHB has been reported within the range of a few hours (Marosi et al., 2016; Sleiman et al., 2016). Our results would be in line with such a course, subsequently manifested by increased protein concentrations, initially detected at the precursor proBDNF. In further support of this view, we observed an association on an individual level between the increase in BHB during the first two hours and the 4-hours increase in proBDNF. As a chance finding cannot be excluded due to the multiple timeframes used as outcomes, these results need replication before conclusions on causality can be made. Also, the double roles of proBDNF—by being a precursor for mBDNF, as well as an active substance in itself—need to be better understood.

Interpretations of the mBDNF response must start by acknowledging that baseline levels were significantly lower in *lowK* compared to *highK*, demonstrating the lower repeatability observed for mBDNF. Consequently, the subsequent differences—with a higher increase in *lowK* vs. *highK*—could be just an illustration of *regression to the mean* (Davis, 1976). If the association was due to a true effect, potential mechanisms include caffeine (Costa et al., 2008; Reyes-Izquierdo et al., 2013) and dietary fat (Genzer et al., 2016), which can stimulate increased levels of BDNF, and could potentially explain the mBDNF increase in *lowK*. To understand why a similar increase was not observed in *highK*, we can only speculate. It should be acknowledged that a phase shift in the circadian rhythm of BDNF mRNA expression has been reported in mice following a very-high-fat ketogenic diet, compared to a non-ketogenic high-fat diet or a low-fat diet (Genzer et al., 2016). If ketosis constitutes an underlying mediator of those findings, circadian interference should not be excluded as a potential confounder in our study. Nevertheless, the absence of an association between BHB and mBDNF on an individual level, except at T0, makes us prone to interpret the mBDNF changes as caused by chance, or by factors unrelated to BHB. Such factors might include differing effects of the fatty acids on proteins regulating the conversion from proBDNF to mBDNF, as reported by Müller et al. (2003). However, the applicability of those findings to our study context may be limited, although our observations of a significantly higher rise in the mBDNF/proBDNF ratio during the first two hours for *lowK* vs. *highK* would be in line with a mechanism related to conversion between the two BDNF forms. The positive association between BHB and mBDNF at baseline calls for replication in wider BHB ranges and is interesting in the light of reported associations between BDNF and memory performance in older adults (Shimada et al., 2014; Mizoguchi et al., 2020), as well as in other populations and neurological or psychiatric conditions (Lima Giacobbo et al., 2019). Increased mBDNF has further been reported after two weeks on a ketogenic diet, subsequently returning to baseline levels (Mohorko et al., 2019). Improved BDNF function could potentially constitute one mechanism by which ketogenic strategies, e.g., carbohydrate restriction, time-restricted feeding and ketogenic supplements, could affect brain health. However, the moderate repeatability of mBDNF stresses the importance of replication and reporting of potential null-findings for better understanding of its relation to lifestyle and health.

For proBDNF, the repeatability (i.e., ICC) of baseline levels from the different study days was excellent while the more well-studied biomarker mBDNF showed higher variability within individuals. The literature on BDNF is dominated by studies on mBDNF, or totBDNF as many studies have not discriminated between mBDNF and proBDNF. In our data totBDNF correlated almost perfectly (*r* = 0.98) with mBDNF, and although this could vary depending on the laboratory assay, the impact of proBDNF appears largely underexplored compared to mBDNF. Some researchers have promoted the incorporation of proBDNF for diagnostics, e.g., for affective disorders (Hashimoto, 2015), and our results indeed encourage further studies on its potential association with various diagnoses or cognitive performance. Our interpretation of ICC as a measure of biomarker validity, rather than assay accuracy, is based on satisfactory low CV%-values for both intra- and inter-assay precision (CV% for mBDNF was even slightly better than for proBDNF) together with highly controlled sampling, storage and thawing.

The interpretation of BDNF levels in terms of good/bad/neutral may not necessarily be the same for steady-state vs. dynamic changes, for brain vs. periphery, or for different stages of disease. But recent observations in human post-mortem samples from AD patients suggest that serum BDNF levels in some aspect might reflect brain function: Higher levels of proBDNF in serum correlated with lower proBDNF levels and higher pTau staining in hippocampus (Bharani et al., 2020). Moreover, as the same authors found lower proBDNF levels in the entorhinal and frontal cortices of AD cases compared to controls, as well as differences in other markers of the BDNF pathway between AD and controls, they concluded that a link between BDNF function and AD pathology is supported (Bharani et al., 2020). Interestingly, Bharani et al. (2020) additionally reported that proBDNF levels may be more sensitive than mBDNF or totBDNF to inflammation and AD pathology. Their findings imply that proBDNF could be a promising target for further studies as a diagnostic or a disease monitoring tool in AD, and that is further supported by the indications of superior repeatability for proBDNF over mBDNF in our data.

A limitation of this study is that it was not primarily designed to analyze BDNF responses. The range of ketosis was relatively low for that purpose (Marosi et al., 2016), and the sample size may have provided insufficient power for the analyses. Future studies may also consider parameters like: 1. Neural activity, since action of BHB has been observed particularly on activity-dependent *bdnf* promotors (Sleiman et al., 2016); 2. Serum vs. plasma, as diverging responses has been reported, e.g., after physical exercise in older adults (Nilsson et al., 2020); 3. A longer time span to capture delayed effects and diurnal rhythms. Further, it would be informative to include measures of proteins regulating the conversion from proBDNF to mBDNF, e.g., tissue plasminogen activator (tPA) (Pang et al., 2004). The detection of potential acute effects on cognitive performance in correlation with peripheral BHB and BDNF changes could support the assumption that these peripheral biomarkers are related to brain function, but the tight logistics of the current study unfortunately did not allow the inclusion of a cognitive test. Although BHB levels were assumed to constitute the main difference between the arms, we cannot exclude the influence by other differing factors between the test oils. Importantly, even under the assumption that the reported differences within this study represent causal mechanisms, the study design does not allow conclusions on whether the differences arise from changes in synthesis, stability, utilization, or redistribution between BDNF pools. The generalizability from this small sample of healthy older adults may be limited. But, considering the wide range of mBDNF in the sample—overlapping e.g., the heterogenous ranges reported from samples of Alzheimer patients (Xie et al., 2020)—we consider the direction of the ICC results fairly general. A strength of our study is the distinction of proBDNF and mBDNF, and the well-controlled conditions for collection of complete data. In this article we report comparisons both by arms and on an individual level, and also for different timeframes. Whereas the absence of *one* prespecified outcome variable may be a limitation, it should be noted that the implied delayed association between BHB and proBDNF is supported both by comparisons on an individual level and by comparisons between arms, even with a Bonferroni adjusted threshold for the *p*-value due to using four different timeframes.

The potential of using ketogenic supplements as a complemental strategy to carbohydrate restriction is discussed as ketogenic diet therapies are increasingly investigated in conditions beyond epilepsy (Kossoff and Cervenka, 2020), which include MCI/AD (Krikorian et al., 2012; Taylor et al., 2018; Brandt et al., 2019; Nagpal et al., 2019; Neth et al., 2020; Phillips et al., 2021). In a previous publication from the current clinical trial (Norgren et al., 2020b), we demonstrated that supplementation with kMCT and time-restricted feeding regarding carbohydrates provide two—optionally additive—strategies to induce transient ketosis in older adults following their normal diet. Herein, we further provide preliminary evidence that the induced ketosis may be related to altered serum levels of a protein essential for cognitive function. Whether such changes, alongside improved brain energetics (Cunnane et al., 2020), would translate into long-term effects on cognitive health requires further studies.

In summary, we observed an association between change in BHB and change in proBDNF with a few hours delay, which would be in line with a signaling role of BHB as observed in non-human studies. However, replication is needed before conclusions can be made on the impact of ketosis on serum BDNF in humans. The positive association between mBDNF and BHB at baseline warrants follow-up in wider BHB ranges. Unfortunately, interpretations of the subsequent changes in mBDNF were impeded by large intra-individual variability in baseline levels. The precursor proBDNF was superior to mBDNF regarding repeatability and inter-individual discriminability. Future studies on proBDNF as an intervention or disease monitoring biomarker should be encouraged.

## Data Availability Statement

The datasets presented in this article are not readily available because of legal restrictions to only make data available to appropriate researchers. On reasonable request, the data will be made available by the authors provided this can be done without conflicting ethical or legal regulations. Requests to access the datasets should be directed to Anna Sandebring-Matton, anna.matton@ki.se.

## Ethics Statement

The studies involving human participants were reviewed and approved by The Regional Ethical Review Board in Stockholm. The participants provided their written informed consent to participate in this study.

## Author Contributions

JN, SS, UA, KN, SR, TN, MK and AS-M conceived and designed the intervention. JN, AS-M and MD developed this sub-study. KN performed the intervention. MD and AS-M performed the BDNF laboratory analyses. JN analyzed data and interpreted the results together with MD, IK, and AS-M who also contributed to the first version of the manuscript. JN prepared figures and drafted manuscript. All authors edited and revised the manuscript, and approved the final version.

## Conflict of Interest

The authors declare that the research was conducted in the absence of any commercial or financial relationships that could be construed as a potential conflict of interest.

## Publisher's Note

All claims expressed in this article are solely those of the authors and do not necessarily represent those of their affiliated organizations, or those of the publisher, the editors and the reviewers. Any product that may be evaluated in this article, or claim that may be made by its manufacturer, is not guaranteed or endorsed by the publisher.
